# Floating Subscapularis Tear: A Variation of the Partial Subscapularis Tear

**DOI:** 10.3390/jfmk5010011

**Published:** 2020-02-05

**Authors:** Kotaro Yamakado

**Affiliations:** Department of Orthopaedics/Sposrts medicine, Fukui General Hospital, Fukui 9108561, Japan; yamakadok@gmail.com

**Keywords:** arthroscopy, partial thickness rotator cuff tear, subscapularis tear, variation

## Abstract

A variation of subscapularis tear has been identified, named floating subscapularis, where the tendon is completely detached from the lesser tuberosity but is continuous with the tissue covering the bicipital groove. An accurate diagnosis can be made using arthroscopic observation with passive external and internal rotation of the affected shoulder, which shows mismatched movement between the humerus and the subscapularis tendon. The purpose of this study is to examine the prevalence of this particular tear pattern. Clinical records during the study period (from January 2011 to December 2017) were retrospectively examined. Overall, 1295 arthroscopic rotator cuff repair procedures were performed. Among these, the subscapularis tendon was repaired in 448 cases, and 27 cases were diagnosed as floating subscapularis. The prevalence of floating subscapularis was 6% in the subscapularis repair population. This particular tear pattern has not previously been described and it seems to be ignored. The floating subscapularis is thought to be the tear of the deep layer preserving the superficial layer connected to the greater tuberosity by fibrous extension of the soft tissue covering the bicipital groove.

## 1. Introduction

The ball-and-socket structure of the shoulder joint allows a greater range of motion than other joints. The rotator cuff muscles hold the joint for stability and rotate the humerus for mobility. Rotator cuff muscles consist of four muscles: the supraspinatus, infraspinatus, teres minor, and subscapularis. The subscapularis, an internal rotator of the shoulder, arises from the subscapularis fossa of the scapula and inserts into the humerus. The superior two-third part (the tendinous part of the subscapularis) inserts into the lesser tuberosity and the inferior one-third part (the muscular part of the subscapularis) inserts directly into the humerus below the lesser tuberosity by a thin membranous structure [1,2,3].

**Figure 1:**
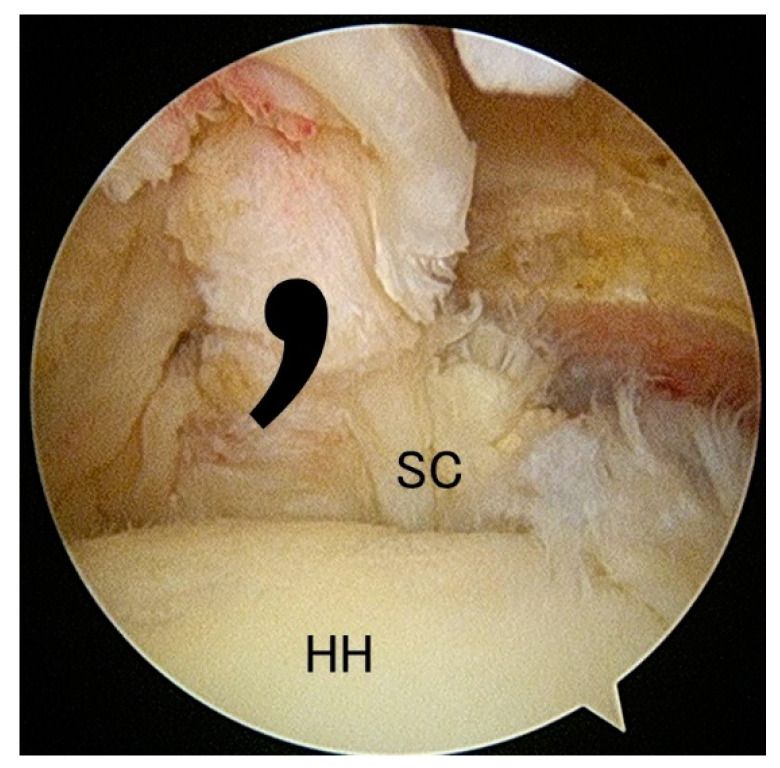
Subscapularis tear in the rt. shoulder. As the superior gleno humeral ligament and coracohumeral ligament are attached to the subscapularis, it assumes a comma shape (so-called “comma sign”), which is thought to be pathognomonic of a subscapularis tear. SC: subscapularis, HH: humeral head.

Tears of the subscapularis tendon are less common than those of the posterosuperior rotator cuff muscles (supraspinatus and infraspinatus). The prevalence of subscapularis tendon tears had been largely underestimated before arthroscopic examination became popular in the 2000s. Currently, the diagnosis of full-thickness subscapularis tear is routinely made arthroscopically and with reliable accuracy, with reporting tear-rate up to 29% [4,5,6]. In most cases of a full-thickness subscapular tear, the torn tendon retracts medially and inferiorly; however, as the superior glenohumeral ligament and coracohumeral ligament are attached to the subscapularis, it assumes a comma shape (so-called “comma sign”), which is thought to be pathognomonic of a subscapularis tear (Figure 1) [7].

Classification of rotator cuff tears can be made according to the tear morphology: acute or traumatic/degenerative, partial or complete, retracted or not retracted, combined or isolated. For subscapularis tears, various classifications have been proposed. But the Lafosse classification [8] is widely accepted. The Lafosse classification provides five grades of tear: (I) Partial lesion of superior third of the subscapularis without tendon retraction; (II) complete tear of the superior one-third; (III) complete tear of superior two-third; (IV) complete tear of subscapularis with tendon retraction and a concentric glenohumeral joint; (V) complete tear of subscapularis with tendon retraction and an eccentric glenohumeral joint. Garavaglia et al. [9] subdivided grade I, distinguishing minor fraying of the insertion site (grade IA) and partial tears of the deep layer (grade IB).

**Figure 2:**
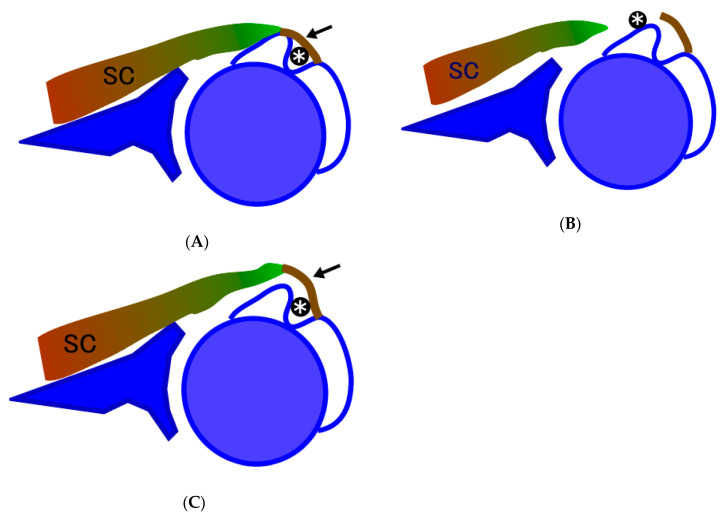
Schematic drawing of normal, torn, and floating subscapularis. (**A**) Intact subscapularis. SC, subscapularis; arrow, transverse humeral ligament; *, long head of the biceps tendon. (**B**) Torn subscapularis. The tendon is detached from the lesser tuberosity and is retracted medially. In many cases, long head of the biceps is subluxated. (**C**) Floating subscapularis. Subscapularis tendon is detached from the lesser tuberosity with soft tissue extension to the transverse humeral ligament is preserved. SC, subscapularis; arrow, transverse humeral ligament; *, long head of the biceps tendon.

**Figure 3:**
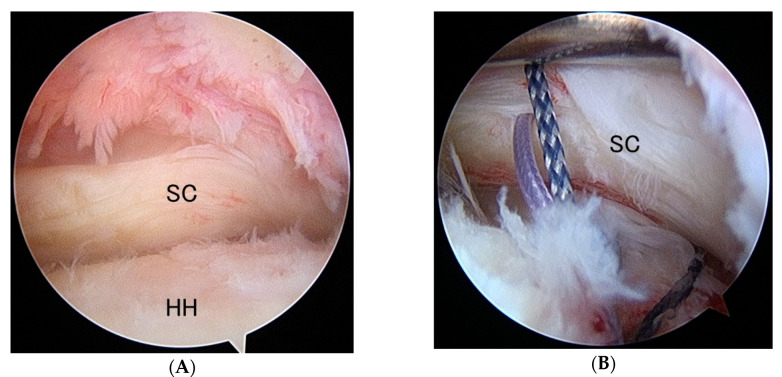
Floating subscapularis. (**A**) Rt. shoulder viewing from the posterior portal. Subscapularis is detached from the lesser tuberosity but is not retracted. The comma structure is preserved and the subscapularis is appearing to be normal at a glance. (**B**) Internal rotation of the shoulder discloses the detachment of the subscapularis from the lesser tuberosity. A suture anchor is inserted on the lesser tuberosity and sutures are applied on the torn subscapularis. SC: subscapularis, HH: humeral head.

**Figure 4:**
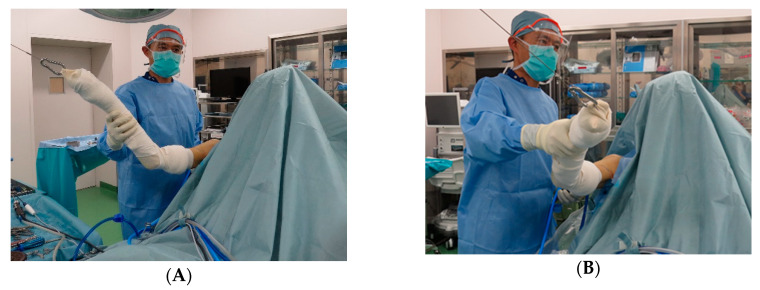
Manipulation methods for identification of the floating subscapularis. Flexion-external rotation (**A**) and flexion-internal rotation (**B**) of the shoulder shows unsynchronized movement of the subscapularis and gap between the subscapularis tendon and the lesser tuberosity.

A variation of subscapularis tear was identified and named as “floating subscapularis,” where the tendon is completely detached from the lesser tuberosity but is continuous with the tissue covering the bicipital groove (Figure 2 and Figure 3). An accurate diagnosis can be made using arthroscopic observation with passive external and internal rotation of the affected shoulder (Figure 4), which shows mismatched movement between the humerus and the subscapularis tendon. The purpose of this study was to examine the prevalence of this particular tear pattern.

## 2. Materials and Methods 

A retrospective review was initiated with the Institutional Review Board approval (31 July 2019). Clinical records during the study period (from January 2011 to December 2017) were retrospectively examined. Demographics of the patient data, associated injury such as long head of the biceps instability, or concomitant cuff tears were reviewed.

### Surgical Technique

The patient was positioned in a beach chair position, with the arm held in flexion and 1–3 kg of longitudinal traction in accordance with the patient’s body weight. The shoulder was widely draped, providing free access below the axillary fold to the inferior angle of the scapula. The superficial anatomy of the shoulder was identified, and the skin was marked to outline the clavicle, acromion, scapular spine, coracoid process, and lateral border of the scapula. The arthropump (ConMed Linvatec, Largo, FL, USA) was turned on, and the pressure was set to 50 mm Hg. Routine arthroscopy portals were used (posterior, lateral, anterior, and anterolateral), and diagnostic arthroscopy using a 30° arthroscope was initiated using a standard posterior portal. 

Viewing from the posterior portal, the subscapularis and the long head of the biceps tendon (LHBT) were observed and examined. In case of the floating subscapularis tear pattern, torn tendon was not retracted and the comma sign was negative, however, by passive rotation and external rotation, movement of the subscapularis unsynchronized with the movement of the humerus (Figure 4). If the pulley of the LHBT was torn, LHBT was subluxated between the lesser tuberosity and subscapularis by passive external rotation of the shoulder. If the LHBT pulley was intact, the LHBT was looked stable but was shifted more medially and removing the pulley revealed the exposed lesser tuberosity. Once an accurate diagnosis was made, arthroscopic repair was done using the suture anchor technique. Postoperatively, the patients were immobilized with an abduction pillow for four weeks. Passive shoulder range of motion exercises were initiated postoperatively from the first postoperative day. Strengthening exercises began 12 weeks postoperatively. 

## 3. Results

Overall, 1295 arthroscopic rotator cuff repair procedure were performed. Among these, subscapularis tendon was repaired in 448 cases, and 27 cases (6%) were diagnosed as floating subscapularis. The mean age of the patients was 66.3 years (Table 1). There were two isolated subscapularis tears, 4 combined with partial supraspinatus tear, 11 combined with full-thickness supraspinatus tear, and 10 with full-thickness supraspinatus and infraspinatus tears. Fifteen out of 27 cases (56%) had LHBT pathology: 9, full tear; 6, partial tear; 2, hypertrophy; 7, subluxation from the bicipital groove.

## 4. Discussion

The prevalence of floating subscapularis was 6%. This particular tear pattern has not previously been described; it has either been ignored or overlooked. The clinical presentation for the subscapularis tears including the floating subscapularis tear were similar to the common rotator cuff disease. A high index of suspicion for subscapularis tears should be held. Patients complain pain, particularly overhead work, typically exacerbates in repetitive or heavy use. Tenderness over the bicipital groove was common. This symptom could be related to the long head of the biceps pathology highly concomitant to the subscapularis tears. Difficulty with activities such as washing the body or holding down the goods on the table seemed to be characteristic manifestations of the subscapularis tears (the “washing body sign”). Clinical tests including the belly press test, the lift-off test, and the bear-hug test were highly positive; however, unlike the full thickness subscapularis tears, passive external rotation was not increased in the floating subscapularis tears.

There were several variations of partial or full thickness subscapularis tear in which visualization and/or diagnosis is difficult even with arthroscopic observation [10,11,12]. Walch et al. described the “hidden lesion” of the subscapularis where the presence of an intact biceps pulley or rotator interval hiders the visualization of subscapularis tear during open surgery [10]. Neyton et al. emphasized the difficulty even with the advancement of arthroscopic technique [12]. In our series, the pulley appeared normal in 3 of the 14 cases (21%). It appears that the floating subscapularis overwrapped highly with the hidden lesion and complete visualization by removing the pulley might be necessary for complete visualization of the lesion. Yoo et al. proposed a subscapularis tear classification based on the tear morphology [11]. They described a tear pattern that looked like partial tear at first (fraying or longitudinal split of the leading edge of the subscapularis) from the intra-articular arthroscope view but turned out to be an intratendinous tear after removing or retracting the biceps away from the groove. They named this variation the “concealed tear.” This variation is different from the floating subscapularis as the concealed tear is an intratendinous tear which spares joint side insertion of the subscapularis.

The LHBT passes through the bicipital groove and lies between the lesser and treater tubercles of the humerus. The tissue covering the bicipital groove is called the transverse humeral ligament. Singh and colleagues have shown that this structure is not a ligament but the tendinous extension from the subscapularis, supraspinatus, or pectoralis minor fibers [13]. Tabaa et al. reported a study of histological structure of the subscapularis tendon from its humeral insertion point to the musculotendinous junction [14]. They showed that the subscapularis tendon is composed of two distinct fibrous layers. The superficial layer of the subscapularis tendon passes across the bicipital groove and forms a fibrous ring around the LHBT that stabilizes the latter in the bicipital groove. We think that the floating subscapularis is the tear of the deep layer preserving the superficial layer connected to the greater tuberosity by fibrous extension of the soft tissue covering the bicipital groove (Figure 2c and Figure 3).

## 5. Conclusions

A variation of subscapularis tear, named floating subscapularis, was identified. Accurate diagnosis can be made by arthroscopic observation while performing passive external and internal rotation of the affected shoulder. If there is a tear, the movements of the humerus and the subscapularis tendon will be unsynchronized. All authors have read and agreed to the published version of the manuscript.

## Figures and Tables

**Table 1:** Demographics.

Variable *			
Age, y		66.3 (7.6)	
Gender			
	Male	22	
	Female	11	
Dominant side		59%	
ROM (active)			
	Forward flexion, °	122 (43)	0 to 170
	ER at side, °	49 (21)	10 to 80
UCLA score		17.3 (4.8)	5 to 25
VAS (pain), mm		65 (19)	
Associated cuff tears			
	Partial tear of the supraspinatus	4	
	Full-thickness tear of surpaspinatus	11	
	Full-thickness tear of surpaspinatus and infraspinatus	10	
Status of the long head of biceps tendon			
	normal	3	
	hypertrophy	2	
	Partial tear	6	
	full tear	9	
	subluxation	7	

* Continuous data are presented as the mean ± standard deviation (range) or as indicated, and categoric data as number (%) or number. Values are expressed as mean (SD). Abbreviations: UCLA score, the University of California Los Angels score; VAS, visual analogue scale.
